# miR-142-3p regulates RDH13 to impair trophoblast function via regulating CDH5/LFA-1/L-SELECTIN axis: a novel mechanism and diagnostic/therapeutic for pre-eclampsia

**DOI:** 10.3389/fmed.2026.1760916

**Published:** 2026-03-25

**Authors:** Long Shan, Kun Lu, Yuan Zhang, Xuejiao Tao, Yalan Ma, Shaomin Fen

**Affiliations:** 1 Department of Obstetrics, Gansu Provincial Hospital, Lanzhou, Gansu, China; 2 Department of Cardiovascular Critical Care Medicine, Affiliated Hospital of Gansu Medical College, Lanzhou, Gansu, China

**Keywords:** biomarkers, cell culture, experimental verification, mir-142-3p, preeclampsia, RDH13

## Abstract

**Background:**

miR-142-3p is aberrantly expressed in preeclampsia placentas, but its regulatory mechanisms in trophoblasts remain incompletely understood. Given that miRNAs exert biological effects primarily by targeting the 3’UTR of downstream genes, we prioritized potential downstream regulators of miR-142-3p to elucidate its pathogenic mechanism in preeclampsia. This study aims to elucidate how miR-142-3p modulates trophoblast functions and its clinical significance in preeclampsia pathogenesis using integrated bioinformatics and experimental approaches.

**Methods:**

Public datasets (GSE25906 and GSE10588) and miR-142-3p-related downstream target genes (predicted by the miRWalk database) were retrieved. Differentially expressed genes were identified by WGCNA and machine learning algorithms. RDH13 was identified as a candidate functional mediator of miR-142-3p based on its intersection with miR-142-3p targets, DEGs, and preeclampsia-related module genes. Clinical samples and HTR-8/Svneo cells were used for validation: miR-142-3p mimics/inhibitors and RDH13 siRNA were transfected to construct gain-of-function and loss-of-function models; co-transfection of miR-142-3p inhibitors and RDH13 siRNA was performed to verify the functional dependency. RT-qPCR and WB detected gene/protein expression. Cell apoptosis, invasion, migration, and proliferation were evaluated to clarify the functional roles of miR-142-3p and RDH13 in preeclampsia.

**Results:**

Through bioinformatics analysis, HEXB and RDH13 were identified as potential early biomarkers of preeclampsia diagnosis. Clinically, miR-142-3p was upregulated while RDH13 was downregulated in preeclamptic samples. In HTR-8/Svneo cells, miR-142-3p promoted apoptosis and inhibited invasion, migration, and proliferation, whereas RDH13 exerted opposing effects. Rescue experiments confirmed that RDH13 is a functional downstream regulator of miR-142-3p, and the regulatory roles of miR-142-3p were partially mediated by suppressing RDH13 expression—consistent with canonical miRNA—downstream gene regulatory mechanisms. Importantly, these regulatory roles of miR-142-3p were partially mediated by RDH13, which further coordinately regulated the mRNA and protein expression of CDH5, LFA-1, and L-SELECTIN across experimental groups.

**Conclusion:**

This study identifies miR-142-3p/RDH13 axis as a potential diagnostic biomarker and a promising candidate target for preeclampsia research. Functional and rescue assays confirm that miR-142-3p negatively regulates RDH13, thereby suppressing trophoblast proliferation, invasion, and migration while promoting apoptosis, revealing a novel preeclampsia-related regulatory mechanism.

## 1 Introduction

Preeclampsia, a multisystemic clinical disorder specific to pregnancies beyond 20 weeks of gestation, is characterized by hypertension and proteinuria as its cardinal manifestations; in severe cases, it may be accompanied by complications such as thrombocytopenia, abnormal liver function indices, and renal impairment (1, 2). Globally, the incidence of preeclampsia ranges from 2 to 8%, with a higher prevalence observed in women with a prior history of preeclampsia, advanced-age individuals, patients with diabetes or hypertension, obese subjects, and multiparous pregnant women (3). Currently, the pathogenesis of this disease is closely linked to insufficient trophoblast invasion of uterine spiral arteries: inadequate trophoblast infiltration directly leads to reduced placental blood perfusion, thereby triggering placental ischemia and hypoxia. Under this pathological condition, the placenta releases various inflammatory factors and vasoactive substances, which can induce systemic vasospasm and cause vascular endothelial cell injury, ultimately resulting in typical symptoms, such as hypertension, proteinuria, and dysfunction of other organs (4). At present, the primary early-intervention therapy for preeclampsia involves oral aspirin, which is the only medication recommended by international guidelines for preeclampsia prevention (5). However, the compliance of preeclampsia patients with prophylactic aspirin is quite low, reflecting significant challenges in early pharmacological intervention (6). In light of this, early identification of high-risk pregnant women is a core prerequisite for improving intervention outcomes, and the screening of preeclampsia diagnostic biomarkers with high sensitivity and specificity is of crucial clinical value for achieving early detection and intervention of the disease, thereby reducing the risk of adverse maternal and neonatal outcomes.

MicroRNAs (miRNAs) are a class of non-coding single-stranded RNA molecules that primarily regulate biological processes at the level of gene expression by complementary pairing with the 3′ untranslated region (3’ UTR) of target mRNAs, thereby inducing mRNA degradation or inhibiting its translation (7). Previous studies have demonstrated that aberrant miRNA expression can lead to impaired trophoblast differentiation, increased cell apoptosis, incomplete spiral artery invasion, and reduced placental blood supply in preeclampsia, with severe cases triggering systemic immune responses and oxidative stress within the placenta (8). Among these, miR-142-3p, a miRNA expressed in various human cell types, exhibits altered expression in the placentas of preeclampsia patients, which shows a strong correlation with disease development in early pregnancy (9). More importantly, miR-142-3p regulates the biological functions of trophoblast cells through multiple molecular mechanisms: specifically, it disrupts the TGF-β1/Smad3 signaling pathway and targets the FOXM1 gene, which either individually or collectively inhibits the migration, invasion, and proliferation of trophoblast cells (10, 11). Meanwhile, it can also regulate the NF-κB signaling pathway by targeting the HMGB1 gene, thereby modulating immune and inflammatory responses within the placental microenvironment (11). Ultimately, miR-142-3p not only governs the core biological behaviors of trophoblast cells but also participates in the pathogenesis and progression of pregnancy-related pathological processes, such as preeclampsia and early pregnancy loss. In view of the special role of miR-142-3p in preeclampsia, it is necessary to further explore the functional association between miR-142-3p and its potential downstream mediators in preeclampsia for early diagnosis and treatment of preeclampsia.

To address these knowledge gaps, this study primarily integrates bioinformatics approaches and experimental validations to systematically investigate the regulatory role of miR-142-3p in preeclampsia trophoblast cell biology. Our aim is to elucidate its mechanistic contributions to preeclampsia pathogenesis and identify it as a potential diagnostic biomarker and candidate target for further therapeutic exploration. Clinically, this study not only deepens the understanding of the molecular mechanisms underlying preeclampsia but also provides a theoretical basis for early screening, risk stratification, and targeted intervention of the disease, which is of great significance for improving maternal and fetal outcomes and reducing the global burden of preeclampsia.

## 2 Materials and methods

### 2.1 Data set information

First, the GSE25906 dataset (platform ID: GPL6102) was retrieved from the Gene Expression Omnibus (GEO) database^1^, which comprises placental tissue samples from 23 patients with preeclampsia and 37 normal individuals, and this dataset was designated as the training set in the present study. Concurrently, the GSE10588 dataset (platform ID: GPL2986) was obtained from the same database; this dataset includes placental tissue samples from 17 preeclamptic patients and 26 normal controls, which was utilized as the validation set for subsequent model verification. Furthermore, to identify miR-142-3p-related genes (MRGs), targeted prediction analysis was further performed using the miRWalk database^2^ (12), which is a well-recognized and comprehensive resource. It incorporates more than 12 established prediction algorithms along with both computationally inferred and experimentally validated miRNA-target interactions, thereby mitigating the inherent bias associated with single-algorithm prediction tools. After rigorous screening and validation, a total of 7,695 eligible MRGs were ultimately confirmed.

### 2.2 Identification and enrichment analysis of differentially expressed genes (DEGs)

In the present study, the “limma” (v 3.52.4) package (13) was employed to perform comparative analysis of gene expression profiles between different groups (disease group vs. control group), thereby identifying DEGs across the groups. The screening criteria for DEGs were defined as |log2 fold change (FC)| > 0.5 and a statistical significance level of *p* < 0.05. To intuitively visualize the expression difference characteristics of DEGs, a volcano plot was generated using the “ggplot2” (v 3.3.6) package (14), while a heatmap was constructed with the “heatmap3” (v 1.1.9) package (15) to achieve visual display of the expression patterns of all DEGs. Furthermore, to elucidate the biological functions of the identified DEGs, the “clusterProfiler” (v 4.7.1) package (16) software was utilized to conduct Gene Ontology (GO) functional annotation and Kyoto Encyclopedia of Genes and Genomes (KEGG) pathway enrichment analysis for these DEGs. A *p*-value < 0.05 in statistical tests was set as the threshold to determine statistically significant differences.

### 2.3 Construction of weighted gene co-expression network analysis (WGCNA)

For the GSE25906 gene expression profile dataset, the present study first performed sample clustering analysis to exclude outlier samples, which laid a solid foundation for subsequent reliable network construction. Subsequently, the WGCNA package software was utilized to construct a weighted gene co-expression network. During the network parameter optimization phase, the scale-free fit index (R2) was adjusted to a level close to 0.9, based on which the appropriate soft-thresholding power (*β*) required for network construction was determined, as adherence to the scale-free network property is critical for ensuring biological relevance. In the gene module division step, the dynamic tree-cutting method was employed to cluster genes into distinct modules, with the minimum size of each module set to 30 genes to guarantee that each module possesses potential biological significance. Thereafter, Pearson correlation analysis was conducted to calculate the correlation between each gene module and preeclampsia traits, and a correlation heatmap between each module and preeclampsia was generated based on the analysis results to intuitively reflect their associations. Ultimately, modules meeting the threshold criteria of |correlation (cor)| > 0.3 and *p* < 0.05 were defined as the key modules in this study, and the genes contained in these key modules were designated as key module genes. Subsequently, Venn diagram analysis was performed among MRGs, key module genes, and DEGs, thereby identifying candidate genes that are concurrently involved in miR-142-3p regulation, preeclampsia-related co-expression networks, and differential expression patterns.

### 2.4 Machine learning

Based on the candidate gene set obtained from the aforementioned analyses, this study employed three classic machine learning feature selection algorithms, support vector machine-recursive feature elimination (SVM-RFE), least absolute shrinkage and selection operator (LASSO), and Boruta (a random forest-derived algorithm), to screen and validate key genes associated with preeclampsia. Specifically, the SVM-RFE algorithm, which is built on the support vector machine framework, achieves gene selection by recursively eliminating features with low importance and is well-suited for classification and regression tasks involving high-dimensional biological data. Its implementation was accomplished using the “e1071” (v 1.7–12) package (17). The LASSO algorithm compresses the weights of redundant features by introducing an L1 regularization term, which is primarily utilized for feature selection in linear regression models and can effectively mitigate the risk of overfitting. In this study, parameter optimization and gene selection for this algorithm were realized via the “glmnet” (v 4.1–7) package (18). The Boruta algorithm screens for truly associated features through statistical significance testing by constructing a model that compares the importance of a random feature set with that of the original feature set, and its analytical process was performed using the “Boruta” (v 8.0.0) package (19). After each of the three algorithms completed gene selection independently, Venn diagram intersection analysis was conducted on the core gene set’s output by each algorithm. Through consensus validation across multiple algorithms, potential preeclampsia biomarkers with stable predictive efficacy in this study were ultimately identified.

### 2.5 Diagnostic efficacy evaluation and expression level of biomarkers

To validate the diagnostic value of potential key genes for preeclampsia, the present study constructed receiver operating characteristic (ROC) curves in the GSE25906 and GSE10588 datasets, respectively, using the “pROC” (v 1.18.0) package (20). The diagnostic efficacy of these potential key genes in distinguishing preeclampsia cases from normal controls was quantitatively evaluated by calculating the area under the ROC curve (AUC), where an AUC value closer to 1 indicates superior diagnostic performance. To further verify the reliability of the expression patterns of these key genes, box plots of their expression levels between groups were generated separately using the expression profile data of the aforementioned genes in both the GSE25906 and GSE10588 datasets, enabling visual comparative analysis of gene expression differences between the case and control groups. Ultimately, genes that exhibited consistent expression trends across both datasets and showed statistically significant differences between groups in each were selected and designated as potential preeclampsia biomarkers associated with miR-142-3p.

### 2.6 Cell culture, transfection, and grouping

The HTR-8/Svneo human chorionic trophoblast cells used in this study were purchased from Wuhan PunuoCell Biotechnology Company. Cryopreserved HTR-8/Svneo human chorionic trophoblast cells were retrieved from liquid nitrogen tanks and immediately placed in a water bath at 37 °C for rapid thawing until completely melted. After the thawed cell suspension was transferred to a centrifuge tube, 5 mL of the complete medium containing 10% fetal bovine serum (FBS) was added, followed by centrifugation at 1,000 rpm for 5 min. The supernatant was discarded, and the cells were resuspended in 1 mL of culture medium before being transferred to a T-25 flask. An additional 5 mL of complete medium supplemented with 10% FBS was added, and the flask was incubated in a 37 °C incubator with 5% CO_2_. Twenty-four hours prior to transfection, the cells were seeded into six-well plates at a density of approximately 2 × 105 cells per well. Transfection was initiated when the cell confluency reached 85%: 2 μg of plasmid was diluted in 250 μL of serum-free medium and incubated at room temperature for 5 min. In addition, 5 μL of Lipo3000 was diluted separately in 250 μL of serum-free medium and also incubated at room temperature for 5 min. The two diluted solutions were mixed and allowed to stand at room temperature for 20 min before being added to the cell suspension. The culture plate was gently shaken, and the cells were incubated in a 37 °C, 5% CO2 incubator for 48 h. The HTR-8/Svneo cells were divided into eight groups for the experiment: blank control group, mimics-NC group, miR-142-3p mimics group, inhibitor-NC group, miR-142-3p inhibitor group, si-NC group, si-RDH13 group, and miR-142-3p inhibitor + si-RDH13 group. After collecting cell samples from each group, the cells were washed twice with phosphate-buffered saline (PBS, AX911702, Aoxing), and the liquid was completely discarded for subsequent experiments.

Meanwhile, five human embryonic tissue samples from the normal group and five from the hypertensive group were collected from Gansu Provincial People’s Hospital, which were utilized for the detection of clinical gene and protein expression levels. Patients were involved in the design, conducting, reporting, and dissemination plans of our research. This study was performed in line with the principles of the Declaration of Helsinki. Approval was granted by the Ethics Committee of Gansu Provincial People’s Hospital (protocol code 2025–036 and 28th Feb, 2025).

### 2.7 Reverse transcription-quantitative polymerase chain reaction (RT-qPCR)

Clinical tissue samples and cells from each group were harvested and lysed with 1 mL of TRIzol reagent (cat. No. RN0102; Aidlab Biotechnologies) for total RNA extraction. Subsequently, complementary DNA (cDNA) was synthesized using the SuperScript III RT reverse transcription kit (cat. No. A502; EXONGEN). PCR amplification was performed using the Sybr qPCR mix kit (cat. No. 4472920; ABI-Invitrogen) with the synthesized cDNA as the template. The PCR amplification system was composed as follows: 10 μL of 2 × qPCR mix, 0.4 μL of forward primer, 0.4 μL of reverse primer, 1 μL of cDNA template, and 8.2 μL of ddH_2_O. The PCR reaction parameters were set as follows: an initial pre-denaturation step at 95 °C for 5 min, followed by 40 cycles of denaturation at 95 °C for 10 s, annealing at 58 °C for 20 s, and extension at 72 °C for 20 s. *β*-Actin was selected as the internal reference gene to normalize expression levels across different samples, and the relative expression levels of the target genes were calculated using the 2^−^ΔΔCt method. The specific sequences of each primer are provided in Table 1.

**Table 1:** Specific sequences of the target primer.

Target name	Primer sequences 5′-3′
β-actin-F	TCCTCCTGAGCGCAAGTACTCC
β-actin-R	CATACTCCTGCTTGCTGATCCAC
RDH13-F	ACTTGAACTGGCAGACGAGG
RDH13-R	TGACCAGCAGCCAGAAGATG
miR-142-3P-F	TCGGCAGGTGTAGTGTTTCCTA
miR-142-3P-R	CTCAACTGGTGTCGTGGA
miR-142-3P-RT	CTCAACTGGTGTCGTGGAGTCGGCAATTCAGTTGAGCTCCATAAA
U6-F	ACGATACAGAGAAGATTAGCATGG
U6-R	AAATATGGAACGCTTCACGAA
CDH5-F	CACCACCAGCTACGATGTGT
CDH5-F-R	TCGATCATGGCTGCCATCTC
LFA-1-F	CGACATCATGGACCCCACAA
LFA-1-R	GCGTCACTTTTTGTGGGGAC
L-SELECTIN-F	AGAACAAGGAGGACTGCGTG
L-SELECTIN-R	TAGTTTGTGGCAGGCGTCAT

### 2.8 Western blotting (WB)

Clinical tissue samples and cells from each group were collected, with approximately 2 × 105 cells sorted per group, followed by washing with PBS two to three times and thorough removal of the supernatant. An appropriate volume of lysis buffer was added to the cells, which were then lysed at 4 °C. Subsequent centrifugation at 12,000 rpm for 10 min was performed, and the resulting supernatant was collected. The protein concentration in the supernatant was determined using a BCA protein assay kit (cat. No. AX913053; Aoxing). SDS-PAGE electrophoresis was conducted with a loading amount of 20 μg protein per well, after which the proteins were transferred onto a PVDF membrane upon completion of electrophoresis. The PVDF membrane was blocked on a shaker at room temperature for 1 h, followed by the addition of primary antibodies: rabbit anti-RDH13 antibody (1:1,000 dilution, cat. No. 16067-1-AP; Proteintech), anti-Integrin α1 antibody (1:5,000 dilution, cat. No. 22146-1-AP; Proteintech), anti-Integrin *β*1 antibody (1:5,000 dilution, cat. No. 12594-1-AP; Proteintech), anti-CDH5 antibody (1:1,000 dilution, cat. No. YP-Ab-17075; Uping), anti-LFA-1 antibody (1:1,000 dilution, cat. No. YP-mAb-16827; Uping), anti-L-SELECTIN antibody (1:1,000 dilution, cat. No. YP-Ab-17086; Uping), and rabbit anti-β-actin antibody (1:1,000 dilution, cat. No. AF7018; Affinity). The membrane was incubated with these primary antibodies at room temperature for 1 h. After incubation, the membrane was washed and then incubated with HRP-conjugated goat anti-mouse secondary antibody (1:1,000 dilution, cat. No. C030205; Affinity) diluted in 1 × TBST containing 3% skimmed milk, with continuous shaking at room temperature for another 1 h. Following additional washes, chemiluminescent reagents were added, and the membrane was subjected to exposure and development (CLINX; China). Finally, the grayscale values of the protein bands were measured for subsequent analysis.

### 2.9 Detection of cell apoptotic activity

In accordance with the operating instructions of the Annexin-V-FITC apoptosis detection kit (cat. No. FXP018; Sijiabai Biotechnology), Annexin-V-FITC and propidium iodide (PI) staining solutions were mixed at the specified ratio. Approximately 1 × 10^6^ cells were resuspended in 100 μL of the mixed staining solution, vortexed gently to ensure homogeneity, and then incubated at room temperature for 15 min. Subsequently, 1 mL of HEPES buffer was added, and the mixture was vortexed again. The fluorescent signals of FITC and PI were detected at an excitation wavelength of 488 nm with band-pass filters at 525 nm and 620 nm, respectively, based on which the apoptosis results of cells in each group were analyzed.

### 2.10 Detection of cell invasion ability

Prior to conducting the Transwell assay, 50–100 μL of Matrigel matrix was added to the Transwell inserts, which were then incubated at 37 °C for 1–2 h to allow solidification. Once the matrix had solidified, the prepared cells were resuspended and washed with PBS, after which their density was adjusted to 1 × 10^5^ cells/mL using serum-free medium containing 0.1% bovine serum albumin (BSA). A 200-μL aliquot of this cell suspension was seeded into each Transwell insert, which was subsequently transferred to the corresponding well of a 24-well plate pre-filled with 700 μL of complete medium. The plate was then placed in a 37 °C incubator with 5% CO_2_ for 48 h of culture. Upon completion of the incubation period, the 24-well plate was removed, and the medium in each well was discarded. This was followed by a single wash with 1 mL of PBS. The bottom surface of each insert was then immersed in 10% methanol solution for 30 s to fix the cells, after which residual fixative was removed by rinsing with methanol. Subsequently, the insert bottom was stained by immersion in crystal violet solution for 2 min, and excess stain was removed by repeated washing with purified water until the background was clear. Finally, non-invading cells remaining inside the Transwell inserts were gently wiped away using a cotton swab. Invading cells on the bottom surface of the inserts were observed under a microscope at 100 × magnification, and the number of cells in each field of view was counted to analyze cell-invasive capacity.

### 2.11 Detection of cell migration ability

To perform the scratch assay, several vertical lines were first drawn evenly on the back of a six-well plate using a pen, ensuring that each line traversed all wells completely. Subsequently, cells from each experimental group were harvested and seeded at a density of 5 × 10^5^ cells per well. The six-well plate was then placed in a culture environment for overnight incubation until the cells reached full confluence across the bottom of each well. Once confluent, scratches were made using a 10-μL pipette tip, with the tip held perpendicular to the pre-drawn lines on the back of the plate. After scratching, the wells were rinsed with PBS to remove cells detached by the scratch. Following this, serum-free medium was added to each well, and the six-well plate was returned to the culture conditions for continued incubation. Images were captured at two time points (0 and 24 h post-incubation), and cell migration was evaluated by comparing the images obtained at these different time points.

### 2.12 Detection of cell proliferation ability

The experimental procedures were strictly performed in accordance with the instructions provided with the CCK-8 assay kit. Specifically, 10 μL of CCK-8 solution was added to each culture well containing cells from respective groups, after which the culture system was incubated continuously at 37 °C for 1 h. To eliminate interference from non-specific signals, a blank control well was set up, which contained an equal volume of cell culture medium and CCK-8 solution as the experimental groups, but was not seeded with any cells. Following incubation, the absorbance (OD) value of each well at a wavelength of 450 nm was measured using a microplate reader. To ensure the reliability of the experimental results, each group of measurements was independently repeated three times, and the average OD value of each group was calculated for subsequent data analysis.

### 2.13 Statistical analysis

All statistical analyses of the experimental data in this study were performed using R software (v 4.2.1). To ensure the reliability and reproducibility of the results, all the aforementioned experimental procedures were independently replicated three times. The resulting data were analyzed using SPSS 19.0 statistical software, with quantitative data that followed a normal distribution presented as the mean ± standard deviation (mean ± SD). Comparisons among multiple groups were conducted using one-way analysis of variance (one-way ANOVA), and a statistically significant difference between groups was defined as *p* < 0.05 or *p* < 0.01.

## 3 Results

### 3.1 Analysis of DEGs

**Figure 1:**
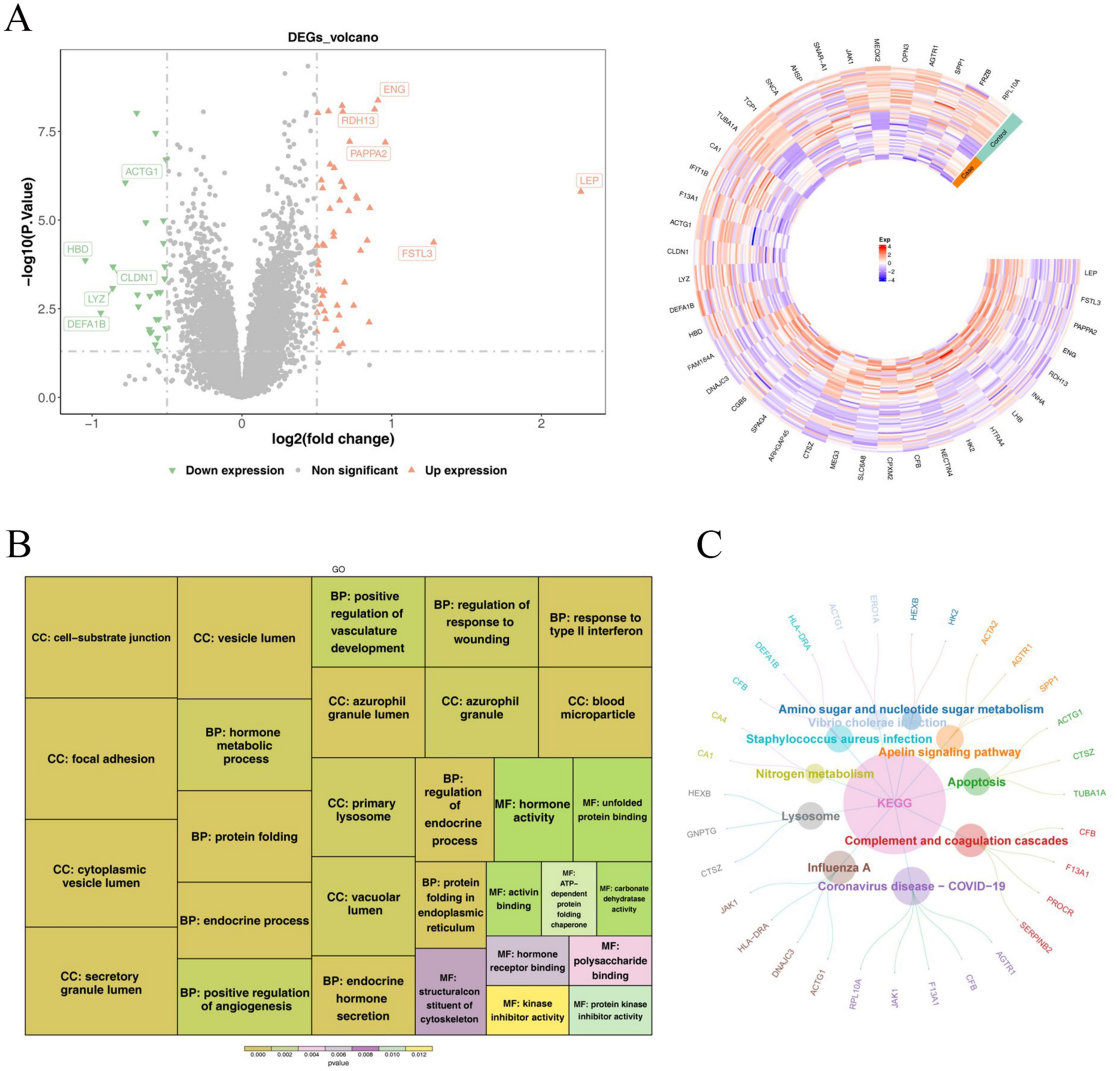
Volcano map and heat map of DEGs **(A)**, GO **(B)**, and KEGG enrichment analysis **(C)**.

Using a significance threshold of *p* < 0.05 for differential screening, the present study conducted a gene expression differential analysis on the GSE25906 dataset, which led to the identification of a total of 77 DEGs. Among these DEGs, 49 were found to be upregulated, and 28 were downregulated, with the heatmap showing distinct clustering of DEGs between disease and control samples, reflecting consistent expression patterns of these genes in each group (Figure 1A). GO functional enrichment analysis revealed that several GO terms, such as positive regulation of angiogenesis, positive regulation of vasculature development, response to type II interferon, endocrine hormone secretion, and hormone activity, were significantly enriched in the DEGs (Figure 1B). These enriched functional terms suggest that the DEGs may be involved in the occurrence and progression of preeclampsia by regulating placental function and maternal vascular health. Furthermore, the results of KEGG pathway enrichment analysis demonstrated that a total of 18 pathways were significantly enriched in the DEGs. These pathways are primarily concentrated in biological processes, such as inflammatory responses, immune responses, and cellular dysfunction, and when combined with the pathological characteristics of preeclampsia, it is hypothesized that such pathways may play a key regulatory role in the pathophysiological mechanisms of preeclampsia (Figure 1C).

### 3.2 Identification of key module genes based on WGCNA

**Figure 2:**
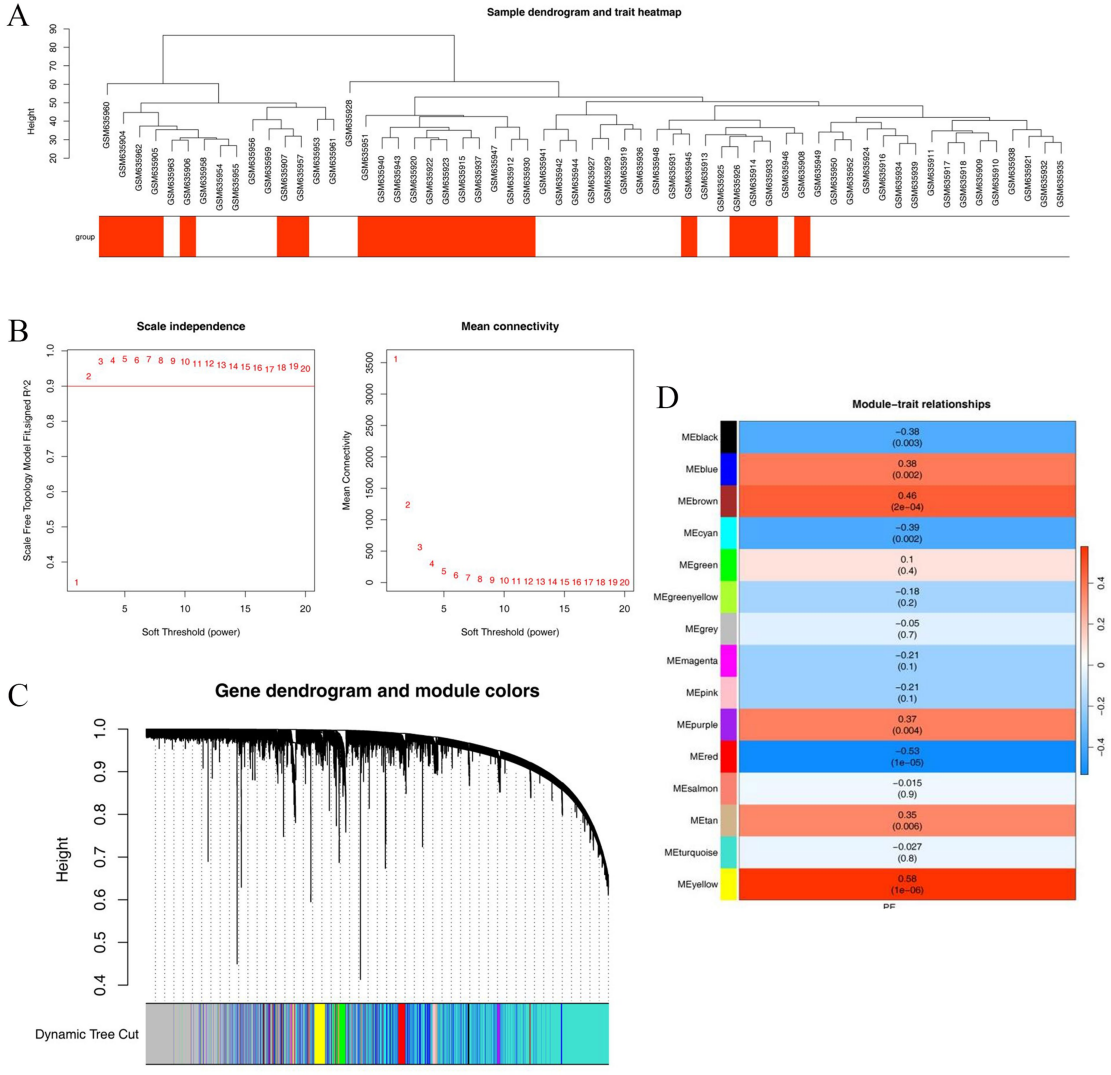
Sample dendrogram and trait heatmap **(A)**, scale independence and mean connectivity **(B)**, dynamic tree cut **(C)**, and module–trait relationships **(D)**.

We performed WGCNA on the GSE25906 dataset. Cluster analysis indicated the absence of outliers, for which no samples were excluded (Figure 2A). The network approximated a scale-free topological structure when the scale-free topology fitting index (R2) approached 0.9, and the average connectivity neared 0 (Figure 2B). Following the merging of dynamic modules, a soft threshold *β* of 9 was set, which led to the identification of 15 modules, as illustrated in the cluster dendrogram (Figure 2C). Subsequent correlation analysis revealed that eight of these modules met the threshold we established for association with preeclampsia, ultimately yielding 4,143 module genes (Figure 2D).

### 3.3 HEXB and RDH13 were screened as biomarkers

**Figure 3:**
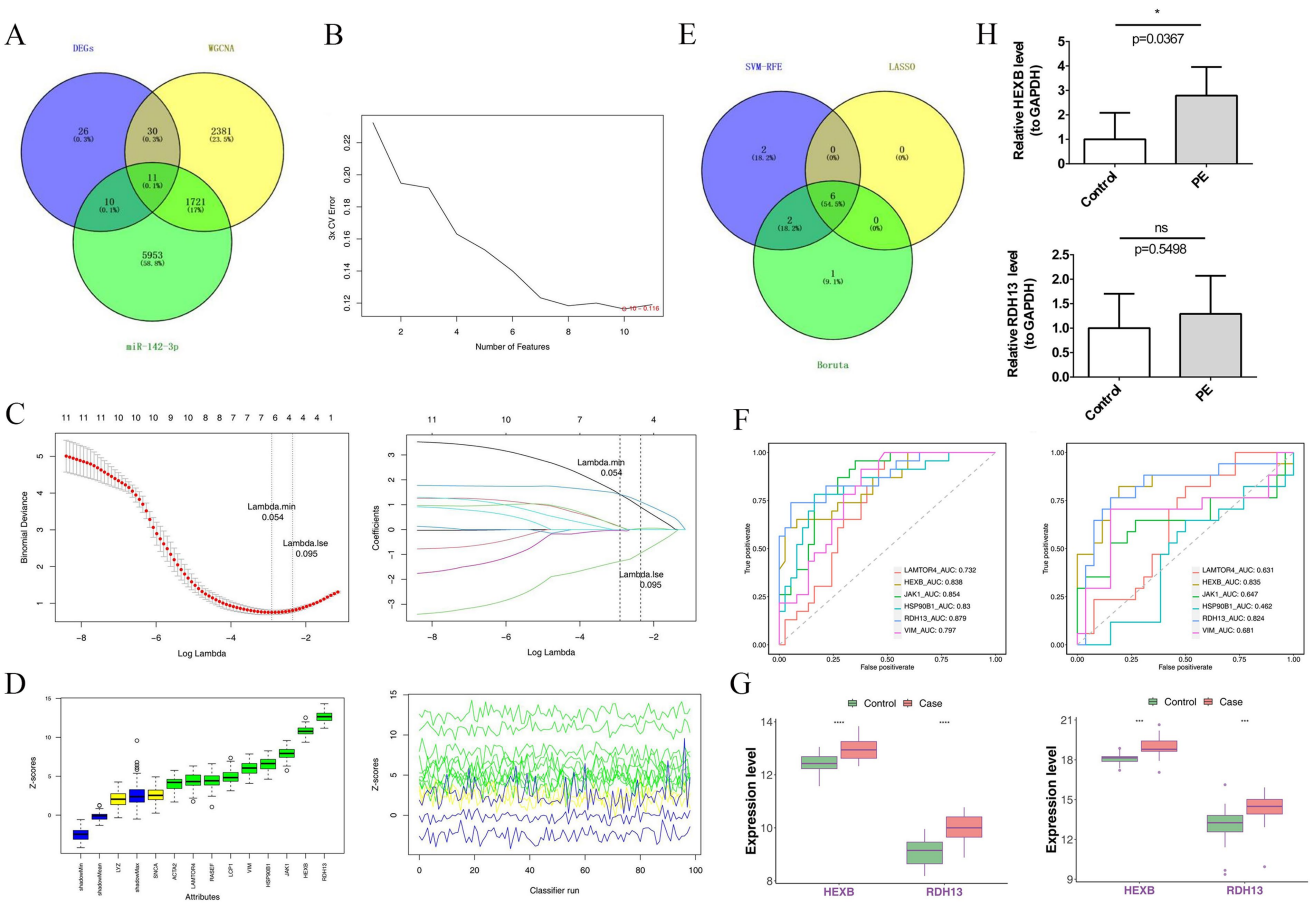
Venn diagram of 11 candidate genes **(A)**, SVM-RFE **(B)**, LASSO **(C)**, Boruta algorithm **(D)** identified hub genes, Venn diagram of potential preeclampsia biomarkers **(E)**, ROC curves **(F)**, box plot of potential preeclampsia biomarkers **(G)**, and mRNA expression levels of biomarkers in RT-qPCR **(H)**. *n* ≥ 3; “ns” indicates *p* > 0.05; “*” indicates *p* < 0.05; “**” indicates *p* < 0.01; “***” indicates *p* < 0.001; and “****” indicates *p* < 0.0001.

First, an intersection analysis was performed on 4,143 module genes, 77 DEGs, and 7,695 MRGs, which resulted in the screening of 11 candidate genes, such as HEXB, LAMTOR4, JAK1, RDH13, RASEF, VIM, SNCA, LCP1, HSP90B1, ACTA2, and LYZ (Figure 3A). Based on this set of candidate genes, three algorithms were employed to screen for hub genes: specifically, the SVM-RFE algorithm identified 10 hub genes (Figure 3B), the LASSO algorithm identified 6 hub genes (Figure 3C), and the Boruta algorithm identified 9 hub genes (Figure 3D). A further intersection of the hub genes obtained from the three algorithms was conducted, ultimately identifying six potential preeclampsia biomarkers, such as LAMTOR4, HEXB, JAK1, HSP90B1, RDH13, and VIM (Figure 3E). To validate the diagnostic value of these genes, ROC curves were plotted using the GSE25906 and GSE10588 datasets. The results showed that the AUC values for both HEXB and RDH13 exceeded 0.8, suggesting that these two genes exhibit favorable diagnostic efficacy in preeclampsia (Figure 3F). Concurrently, box plot analyses were performed to assess the expression levels of the six potential preeclampsia biomarkers in the GSE25906 and GSE10588 datasets (Figure 3G), revealing that only HEXB and RDH13 displayed a significant upregulation trend in preeclampsia samples, with statistically significant differences (*p* < 0.05). Results from clinical samples demonstrated that the mRNA levels of HEXB and RDH13 were also higher in preeclampsia samples, which was consistent with the bioinformatics findings (Figure 3H). Taken together, HEXB and RDH13 were identified as the potential early biomarkers of preeclampsia diagnosis.

### 3.4 miR-142-3p inhibitor suppresses miR-142-3p expression via sequence complementation, and si-RDH13-2 silences RDH13 by targeting its coding region in HTR-8/Svneo cells

**Figure 4:**
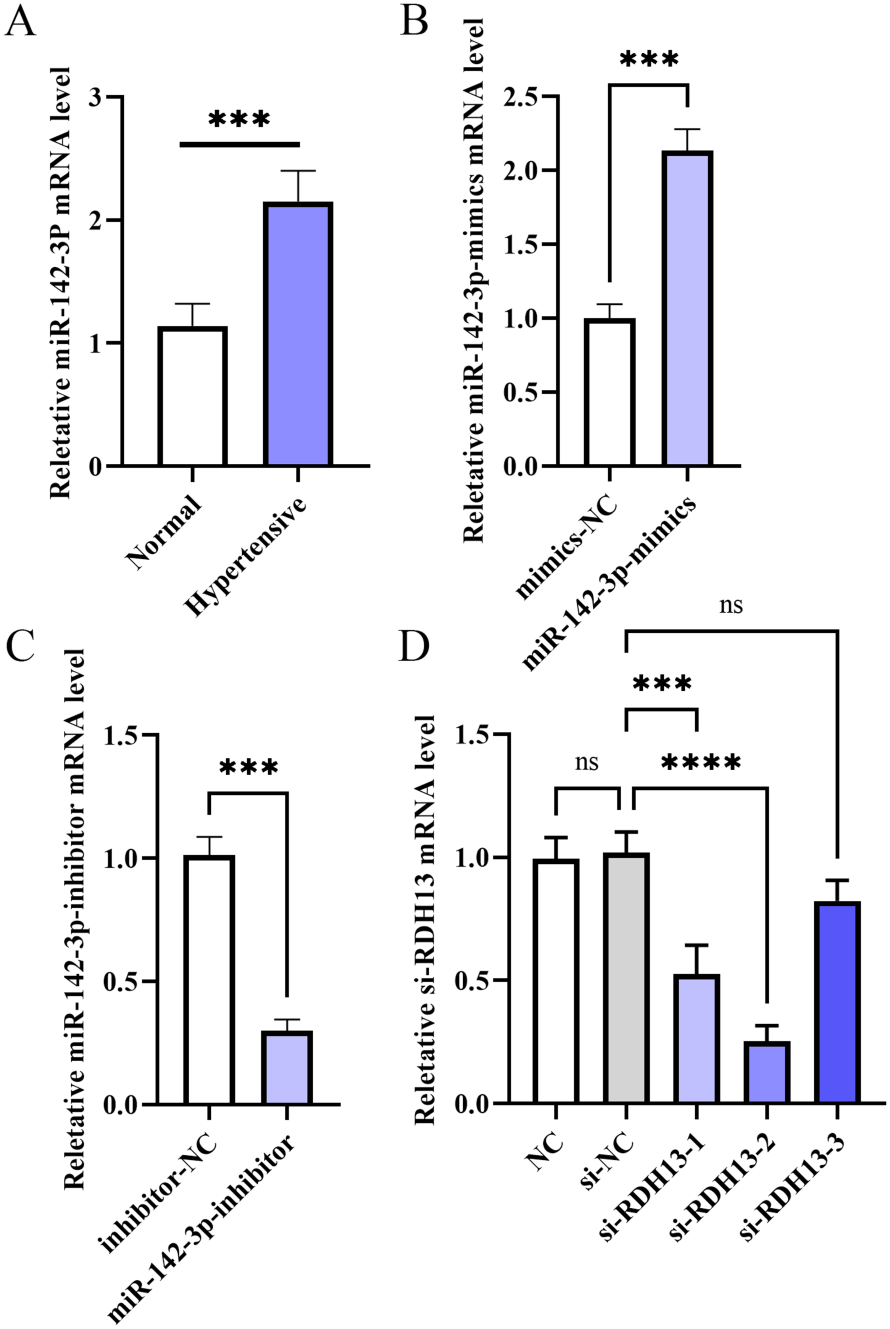
The mRNA expression levels of miR-142-3p **(A)**, miR-142-3p-mimics **(B)**, miR-142-3p-inhibitor **(C)**, and si-RDH13 **(D)**. miR-142-3p upregulation in preeclampsia was consistently validated in clinical samples and two GEO datasets (GSE25906, GSE10588). *n* ≥ 3, “ns” indicates *p* > 0.05; “*” indicates *p* < 0.05; “**” indicates *p* < 0.01; “***” indicates *p* < 0.001; and “****” indicates *p* < 0.0001.

The results of clinical sample detection using RT-qPCR (Figure 4A) demonstrated that, compared with the normal group, the expression of miR-142-3p mRNA was increased in the hypertensive group (p < 0.05). Consistent with this clinical finding, bioinformatics analysis of two independent GEO datasets (GSE25906: 23 preeclampsia vs. 37 normal and GSE10588: 17 preeclampsia vs. 26 normal) further confirmed that miR-142-3p was aberrantly upregulated in preeclampsia placental tissues (p < 0.05), which verified the significant association between miR-142-3p overexpression and preeclampsia occurrence. Mechanistically, the miR-142-3p inhibitor acts as a sequence-complementary oligonucleotide that binds to endogenous miR-142-3p, thereby blocking its ability to interact with target gene 3’UTRs and suppressing its biological function (21). For RDH13 silencing, si-RDH13-2 targets the coding region of RDH13 mRNA, inducing its degradation through the RNA interference (RNAi) pathway—consistent with well-characterized siRNA-mediated gene knockdown mechanisms (22). Additionally, the mRNA expression of miR-142-3p-mimics was higher than that in the control group (Figure 4B), while the mRNA expression of miR-142-3p-inhibitor was lower than that in the control group, which indicated the successful construction of these two vectors (Figure 4C, *P* < 0.05). Finally, since the mRNA expression of si-RDH13-2 was significantly lower than that in other groups, si-RDH13-2 was selected for subsequent experiments (Figure 4D, *P* < 0.05). Notably, the inverse expression pattern of miR-142-3p (upregulated) and RDH13 (downregulated) in preeclampsia clinical samples and GEO datasets, combined with their opposing functional effects in trophoblast cells, supports a negative regulatory relationship between miR-142-3p and RDH13—consistent with canonical miRNA-downstream gene regulatory paradigms (23).

### 3.5 miR-142-3p promotes HTR-8/Svneo cells apoptosis and inhibits invasion, with RDH13 exerting opposing effects

**Figure 5:**
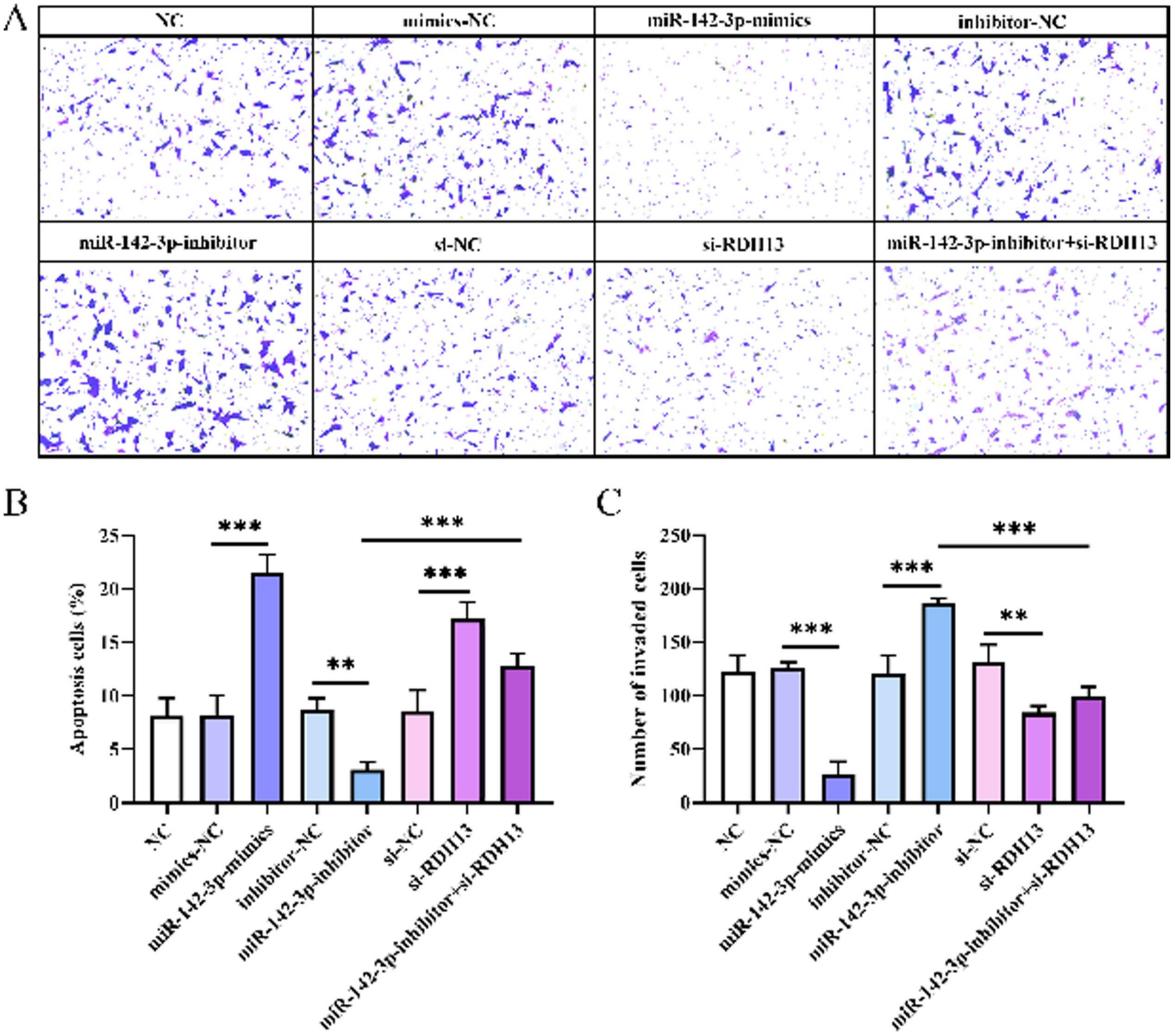
Representative images of HTR-8/Svneo cell invasion detected by Transwell assay **(A)**, apoptosis rate of HTR-8/Svneo cells **(B)**, and number of invaded HTR-8/Svneo cells **(C)**. *n* ≥ 3, “ns” indicates *p* > 0.05; “*” indicates *p* < 0.05; “**” indicates *p* < 0.01; “***” indicates *p* < 0.001; and “****” indicates *p* < 0.0001.

To investigate the roles of miR-142-3p and RDH13 in cell apoptosis, assays were conducted across multiple groups. As shown in Figure 5B, the proportion of apoptotic cells in the miR-142-3p mimics group was significantly higher than in the blank control and mimics-NC groups (*p* < 0.05), while the miR-142-3p inhibitor group displayed a marked decrease in apoptotic cells compared to the inhibitor-NC group (*p* < 0.05). Additionally, the si-RDH13 group increased apoptosis relative to the si-NC group (*p* < 0.05), and co-treatment with miR-142-3p inhibitor and si-RDH13 reduced this pro-apoptotic effect, with fewer apoptotic cells than in the si-RDH13 group alone (*p* < 0.05). These results indicate that miR-142-3p enhances apoptosis, RDH13 suppresses it, and miR-142-3p-mediated regulation of apoptosis is partially dependent on RDH13.

We further assessed their influence on cell invasion. As depicted in Figures 5A–C, the number of invasive cells in the miR-142-3p mimics group was drastically lower than in the mimics-NC group (*p* < 0.05), whereas the miR-142-3p inhibitor group showed a significant increase compared to the inhibitor-NC group (*p* < 0.05). si-RDH13 group reduced invasive cells relative to the si-NC group (*p* < 0.05), and co-treatment with miR-142-3p inhibitor and si-RDH13 reversed this inhibitory effect, with more invasive cells than in the si-RDH13 group alone (*p* < 0.05). These functional phenotypes (apoptosis and invasion regulation) further confirm the inverse regulatory relationship between miR-142-3p and RDH13. Specifically, miR-142-3p overexpression mimics the effect of RDH13 silencing, while miR-142-3p inhibition rescues the pathological phenotypes induced by RDH13 downregulation—supporting that RDH13 is a functional downstream target of miR-142-3p.

### 3.6 miR-142-3p suppresses HTR-8/Svneo cells migration, RDH13 promotes HTR-8/Svneo cells migration

**Figure 6:**
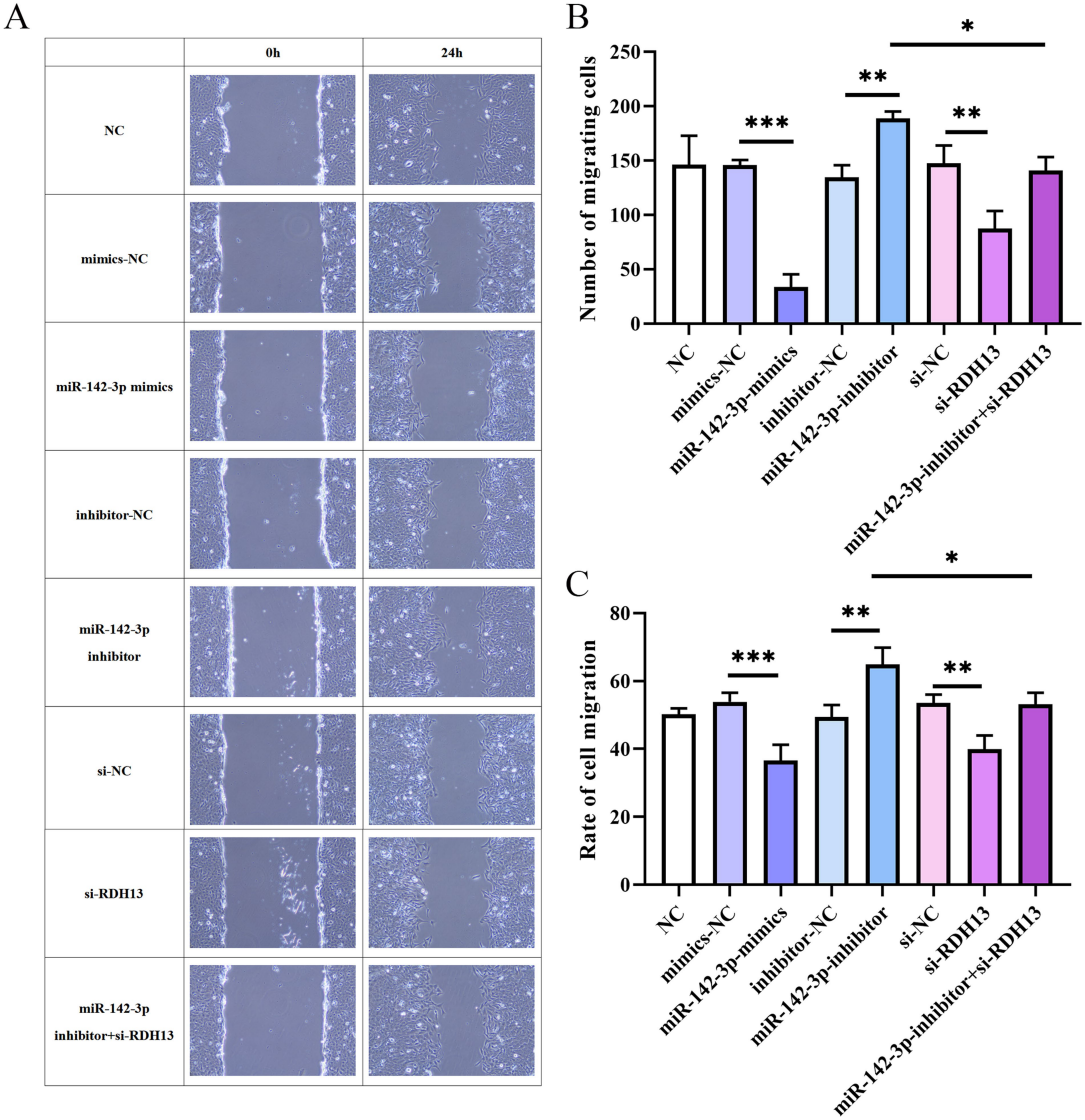
Representative images of HTR-8/Svneo cell migration at 0 h and 24 h detected by wound healing assay **(A)**, number of migrating HTR-8/Svneo cells **(B)**, and migration rate of HTR-8/Svneo cells **(C)**. *n* ≥ 3, “ns” indicates *p* > 0.05; “*” indicates *p* < 0.05; **” indicates *p* < 0.01; ***” indicates *p* < 0.001; and ****” indicates *p* < 0.0001.

Regarding the number of migrated cells (Figure 6B), the miR-142-3p mimics group showed a drastically reduced number of migrated cells compared to the mimics-NC group (*p* < 0.001). Conversely, the miR-142-3p inhibitor group exhibited a significant increase in migrated cell number relative to the inhibitor-NC group (*p* < 0.01). Silencing RDH13 (si-RDH13 group) led to a notable decrease in migrated cell number compared to the si-NC group (p < 0.01). Moreover, co-treatment with miR-142-3p inhibitor and si-RDH13 (miR-142-3p inhibitor+si-RDH13 group) reversed the inhibitory effect of si-RDH13 on cell migration, as evidenced by a significant increase in migrated cell number compared to the si-RDH13 group (*p* < 0.05).

Consistent with these findings, the cell migration rate (Figures 6A–C) exhibited similar trends: the miR-142-3p mimics group had a significantly decreased migration rate compared to the mimics-NC group (*p* < 0.001), while the miR-142-3p inhibitor group showed a marked increase in migration rate relative to the inhibitor-NC group (*p* < 0.01). si-RDH13 group resulted in a substantial reduction in migration rate compared to the si-NC group (p < 0.01), and co-treatment with miR-142-3p inhibitor and si-RDH13 (miR-142-3p inhibitor+si-RDH13 group) reversed this inhibitory effect, with a significant increase in migration rate compared to the si-RDH13 group (*p* < 0.05).

Collectively, these results demonstrate that miR-142-3p suppresses cell migration, RDH13 promotes migration, and the regulatory role of miR-142-3p in cell migration is partially mediated by RDH13.

### 3.7 si-RDH13 significantly reversed the effect of miR-142-3p inhibitor on the proliferation of HTR-8/Svneo cells

**Figure 7:**
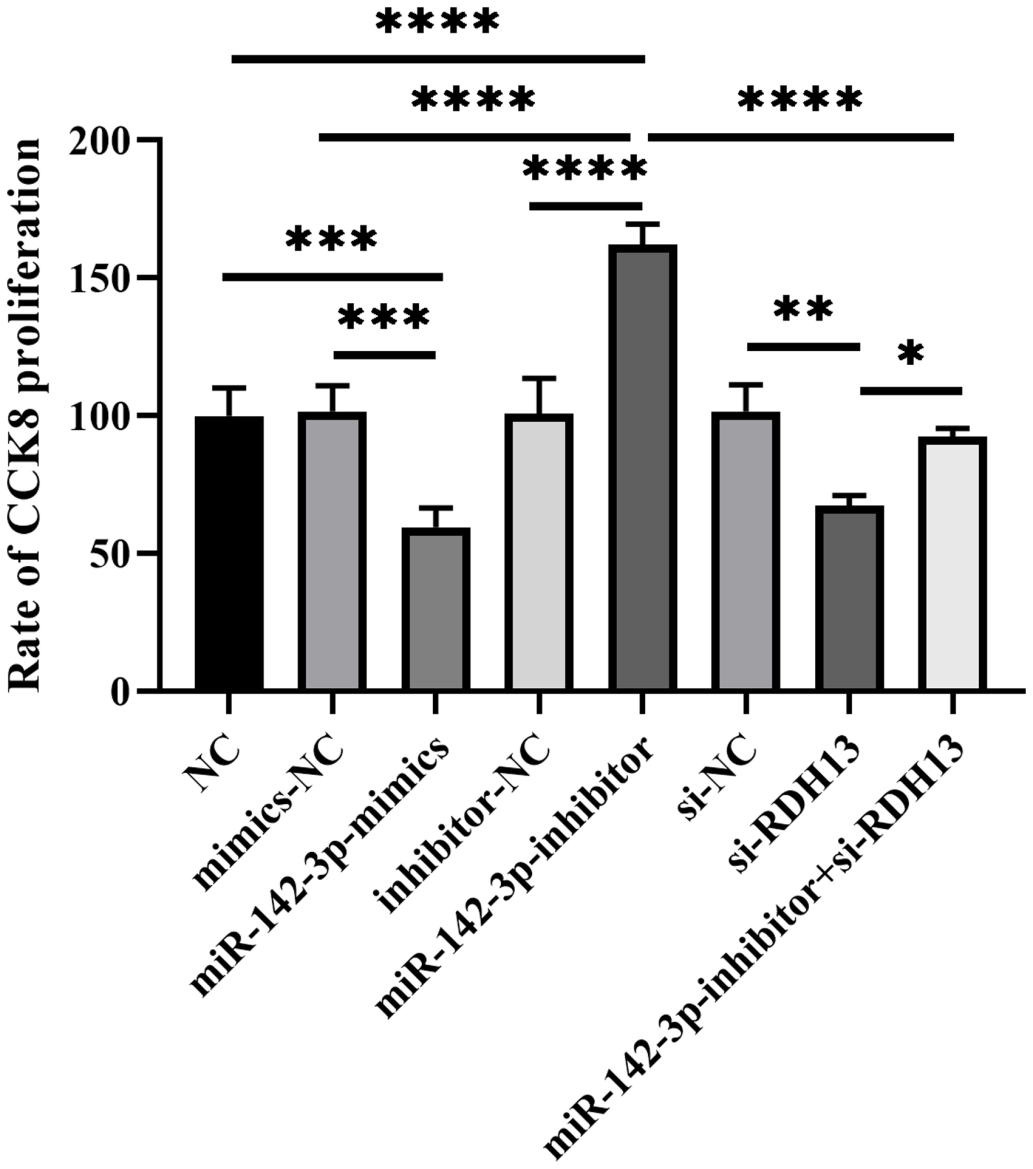
Effect of miR-142-3p and RDH13 on HTR-8/Svneo cell proliferation detected by CCK-8 assay. *n* ≥ 3, “ns” indicates *p* > 0.05; “*” indicates *p* < 0.05; “**” indicates *p* < 0.01; “***” indicates *p* < 0.001; and “****” indicates *p* < 0.0001.

To evaluate the effect of miR-142-3p and RDH13 on cell viability, a CCK-8 assay was performed. As shown in Figure 7, the cell viability in the miR-142-3p mimics group was significantly decreased compared to the mimics-NC group (*p* < 0.001). In contrast, the cell viability in the miR-142-3p inhibitor group was notably increased relative to the inhibitor-NC group (*p* < 0.0001). The si-RDH13 group led to a marked reduction in cell viability compared to the si-NC group (*p* < 0.01). Moreover, co-treatment with miR-142-3p inhibitor and si-RDH13 (miR-142-3p inhibitor+si-RDH13 group) showed a partial recovery in cell viability compared to the si-RDH13 group (*p* < 0.05), although it remained lower than that in the si-NC group. These results demonstrate that miR-142-3p suppresses cell viability, RDH13 promotes cell viability, and the regulatory role of miR-142-3p in cell viability is partially mediated by RDH13. The rescue effect of the miR-142-3p inhibitor on cell proliferation was abrogated by si-RDH13-2. This phenomenon is attributed to the fact that the miR-142-3p inhibitor exerts its pro-proliferative effect by relieving the inhibitory effect of endogenous miR-142-3p on RDH13, while si-RDH13-2 specifically silences RDH13. This directly validates the fact that the regulatory role of miR-142-3p in trophoblast proliferation is mediated through RDH13, further confirming their negative regulatory relationship.

### 3.8 miR-142-3p and RDH13 coordinately regulate CDH5, LFA-1, and L-SELECTIN expression at mRNA and protein levels across groups

**Figure 8:**
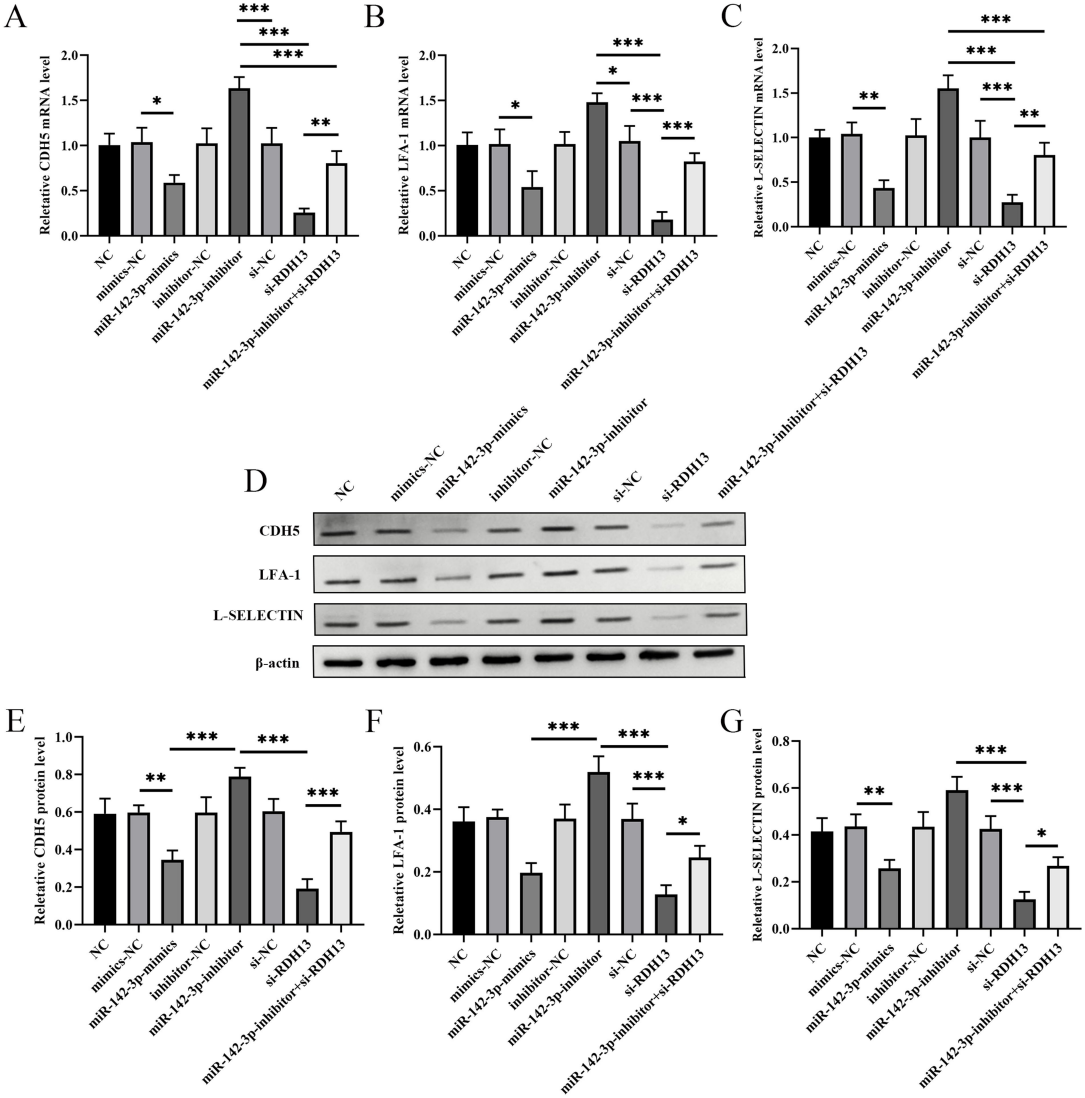
The mRNA expression levels of CDH5 **(A)**, LFA-1 **(B)**, and L-selectin **(C)**, protein level **(D)**, and representative western blotting bands of CDH5 **(E)**, LFA-1 **(F)**, and L-selectin **(G)**. *n* ≥ 3, “ns” indicates *p* > 0.05; “*” indicates *p* < 0.05; “**” indicates *p* < 0.01; “***” indicates *p* < 0.001; and “****” indicates *p* < 0.0001.

The results showed that across eight groups, the mRNA (Figures 8A–C) and protein expression of CDH5, LFA-1, and L-SELECTIN exhibited consistent trends (Figures 8D–G). Original gels are presented in Supplementary Figure 1. Specifically, the miR-142-3p inhibitor group showed significant upregulation of CDH5, LFA-1, and L-SELECTIN at both mRNA and protein levels compared to other groups. In contrast, miR-142-3p mimics and si-RDH13 groups displayed marked downregulation of these molecules at both transcriptional and translational levels. These findings indicate that miR-142-3p and RDH13 coordinately regulate the expression of CDH5, LFA-1, and L-SELECTIN, with consistent patterns observed between their mRNA and protein levels across all groups.

## 4 Discussion

Given that preeclampsia, a severe gestational disorder characterized by trophoblast dysfunction as its core pathological feature, exhibits aberrant expression of the pivotal miR-142-3p in the placenta (9). Our study confirmed that miR-142-3p is significantly upregulated in preeclampsia placental tissues, which was validated by both clinical samples and two independent GEO datasets (GSE25906, GSE10588). This consistent overexpression of miR-142-3p in preeclampsia supports its potential role as a key pathogenic factor, laying the foundation for exploring its downstream regulatory mechanisms. To begin with, two key preeclampsia biomarkers, HEXB and RDH13, were identified and validated through multiple machine learning algorithms combined with ROC curve analysis. Subsequently, to verify the regulatory roles of miR-142-3p and RDH13 in trophoblast cells during preeclampsia, a series of *in vitro* cellular experiments was performed. These experiments demonstrated that in HTR-8/Svneo cells, miR-142-3p promoted apoptosis while inhibiting cell invasion, migration, and proliferation, whereas RDH13 exerted opposite regulatory effects. Collectively, this study simultaneously deepens the understanding of the disease’s pathological mechanisms by elucidating how the miR-142-3p/RDH13 axis modulates trophoblast cell functions and the expression of vascular-related molecules.

In humans, HEXB (*β*-hexosaminidase B), an essential lysosomal enzyme that participates in the degradation of diverse cellular substrates, has been implicated in the pathogenesis of various central nervous system disorders (24). In the present study, we demonstrate that HEXB exhibits elevated expression levels in preeclampsia and possesses substantial diagnostic value, as indicated by an AUC greater than 0.8; this finding aligns with the results of previous investigations, which further enhance the reliability of HEXB as a potential biomarker for preeclampsia (24, 25). Beyond its association with preeclampsia, emerging evidence suggests that HEXB may play a broader role in regulating pregnancy outcomes, as aberrations in the HEXB gene may contribute to the death of PL2074 in pregnancy loss cases. Specifically, as the HEXB gene is localized on chromosome 7, the monosomic abnormality of chromosome 7 observed in PL2074 could directly lead to reduced HEXB expression (26). Given that HEXB-mediated lysosomal enzyme function is critical for maintaining metabolic homeostasis, this insufficient expression may impair glucose metabolism, a process tightly linked to fetal growth and development, thereby disrupting normal developmental trajectories and ultimately resulting in the death of PL2074. Collectively, these observations highlight HEXB’s multifaceted involvement in pregnancy-related pathologies, underscoring its potential as a unifying molecular target for understanding the shared mechanisms underlying preeclampsia and pregnancy loss.

RDH13 (Retinol dehydrogenase 13), a recently identified member of the short-chain dehydrogenases/reductases (SDRs) superfamily, participates in the metabolism of prostaglandins, retinoids, steroids, and aliphatic alcohols (27). Extensive research has explored RDH13 as a diagnostic biomarker for preeclampsia (25, 28), a body of work that aligns with the key findings of the present study, which demonstrate elevated RDH13 expression in preeclampsia. Previous investigations have elucidated that metabolic disturbances in the preeclamptic placenta induce antioxidant defense protein dysregulation and hypoxia, which promote reactive oxygen metabolite accumulation and heightened oxidative stress via the RDH13-mediated dual mechanism-dependent ROS suppression–mitochondrial integrity pathway in hypoxia (29, 30). RDH13 overexpression is proposed as a placental adaptive response that mitigates such damage, though its excessive activation may conversely cause cellular dysfunction and drive preeclampsia progression (31). However, Tsai et al. reported contradictory findings (32). Transcriptomic analysis of human placentas from preeclamptic pregnancies revealed downregulated RDH13 expression, which may be associated with placental dysfunction, immune dysregulation, and placental hypoxia, as these pathophysiological factors collectively contribute to the initiation and progression of preeclampsia (32). Collectively, these divergent findings underscore that the dual roles of RDH13 in preeclampsia are multifactorially determined by the pathological microenvironment and underlying post-transcriptional regulatory networks, highlighting the complexity of its involvement in disease pathogenesis and emphasizing the necessity of considering both its expression levels and functional integrity within the broader pathological landscape of preeclampsia.

Previous studies have consistently demonstrated that impairment of trophoblast cell function within the placenta constitutes a pivotal contributor to the pathogenesis of preeclampsia (33), and consequently, investigating the key regulatory factors that modulate trophoblast cell function not only facilitates a deeper understanding of the physiological and pathological processes underlying placental formation and development but also provides critical insights into the initiation and progression of preeclampsia, findings that may collectively inform the development of novel diagnostic and therapeutic strategies for this complex pregnancy-related disorder. Against this backdrop of RDH13’s complex role in preeclamptic placentas, where its overexpression may reflect both adaptive responses to oxidative stress and potential contributions to pathogenic progression, it becomes critical to explore the regulatory networks that govern its expression and functional output. As we noted earlier, miRNAs have emerged as key post-transcriptional modulators of gene expression in placental development and pregnancy-related disorders (34, 35), with mounting evidence highlighting their capacity to fine-tune trophoblast biology (36, 37). In this context, dysregulated miR-142-3p and RDH13 may form a functional axis that mediates core trophoblast behaviors, including proliferation, apoptosis, migration, and invasion, which processes whose impairment is central to the pathogenesis of preeclampsia, and to test this hypothesis, we performed a series of *in vitro* functional assays in HTR-8/Svneo trophoblast cells (38), with our data providing direct experimental evidence supporting the regulatory interplay between miR-142-3p and RDH13 in modulating these critical cellular processes.

Through a series of systematic in vitro functional assays, the present study uncovers the coordinated regulatory pattern of miR-142-3p and RDH13 in governing trophoblast cell biological behaviors, thereby providing a novel perspective for understanding the pathophysiological mechanisms underlying preeclampsia. Our results demonstrate that miR-142-3p and RDH13 exert opposing effects on trophoblast cell apoptosis, invasion, migration, and proliferation through a functional interplay: transfection with miR-142-3p mimics significantly promoted apoptosis, inhibited invasive and migratory capacities, and reduced cell viability in HTR-8/Svneo cells, while RDH13 silencing via si-RDH13 yielded comparable phenotypic changes. These observations suggest that RDH13 may act as a functional antagonist of miR-142-3p, exerting anti-apoptotic effects and enhancing trophoblast cell invasion, migration, and proliferation. More importantly, co-transfection experiments confirmed that the rescuing effects of the miR-142-3p inhibitor on cell function—including reduced apoptosis and enhanced invasion/migration—were partially abrogated by si-RDH13, a finding that directly validates that the regulatory effects of miR-142-3p are at least partially dependent on RDH13, with the two forming a core axis governing trophoblast cell function. Such miRNA-target gene inverse regulatory axes are not uncommon in placental development; for instance, previous studies have demonstrated that miR-210 and EFNA3 regulate trophoblast cell invasive capacity through a similar mechanism, further supporting the biological plausibility of our findings (39).

From a pathophysiological standpoint, the normal invasion and migration of trophoblast cells are critical for spiral artery remodeling during placental formation, while aberrant apoptosis and defective proliferation are closely associated with the pathological features of placental ischemia and hypoxia in preeclampsia (33). Clinical studies have shown that trophoblast cells in the placental tissues of patients with preeclampsia exhibit significantly reduced invasive depth and increased apoptotic rates, which are highly consistent with the cellular phenotypes induced by miR-142-3p overexpression in the present study (40). The clinical observation that miR-142-3p is highly expressed in the placental tissues of preeclamptic patients, combined with its ability to induce impaired trophoblast cell function in *in vitro* assays, forms a logical loop, suggesting that the dysregulation of the miR-142-3p/RDH13 axis may be a key driver of preeclampsia pathogenesis. Specifically, when miR-142-3p is overexpressed, its inhibitory effect on RDH13 is potentiated, leading to insufficient trophoblast cell invasion, excessive apoptosis, impaired placental development, and ultimately the onset of typical preeclamptic symptoms, such as hypertension and proteinuria (41, 42). The mechanism particularly relevant to early-onset preeclampsia pathogenesis, which is characterized by onset before 34 weeks of gestation, is pathologically dominated by severe placental ischemia–hypoxia and defective spiral artery remodeling—key processes directly regulated by trophoblast function (43). Consistent with this, the miR-142-3p/RDH13 axis’s core role in modulating trophoblast invasion, migration, and apoptosis aligns with the pathological hallmarks of early-onset disease: excessive miR-142-3p would exacerbate RDH13 suppression, further impairing trophoblast infiltration into the uterine decidua and spiral artery remodeling—defects that are more pronounced in early-onset preeclampsia compared to late-onset cases, thus we hypothesize that the miR-142-3p/RDH13 axis may exert a more prominent role in early-onset preeclampsia (44). As previously discussed, RDH13 exerts dual roles in preeclampsia, and while the protective effects demonstrated in functional assays may appear contradictory to the upregulated expression in placental tissues revealed by bioinformatics analyses, this apparent paradox actually confirms the compensatory nature of RDH13 upregulation. Specifically, the oxidative stress microenvironment induced by placental ischemia and hypoxia in preeclampsia (32) can promote the transcriptional upregulation of RDH13 through the activation of pathways such as HIF-1α (27). As a member of the SDR superfamily, RDH13 attempts to alleviate trophoblast cell damage by mediating the “retinal metabolism–ROS scavenging–mitochondrial protection pathway” (30), which involves clearing toxic retinal derivatives and inhibiting mitochondrial ROS outburst, aligning with literature which shows that compensatory gene expression is amplified in severe preeclampsia (45). However, the overexpression of miR-142-3p significantly suppresses the functional exertion of RDH13 through post-transcriptional regulation, rendering this compensatory upregulation insufficient to counteract the damage caused by the pathological microenvironment (46). This ultimately leads to inadequate trophoblast invasion, abnormal apoptosis, and the progression of preeclampsia. This pattern of “compensatory upregulation yet functional limitation” further confirms that the upregulation of RDH13 is not a pathogenic alteration but an adaptive defensive response of the placenta to pathological stress in preeclampsia whose protective potential is blocked by the aberrant high expression of miR-142-3p. Collectively, these mechanistic insights establish a theoretical framework for the future development of targeted therapeutic interventions for preeclampsia via restoration of the miR-142-3p/RDH13 axis homeostasis, such as miR-142-3p antagonism or RDH13 functional potentiation, which necessitates rigorous validation in preclinical *in vivo* preeclampsia models and large-scale multicenter clinical trials to facilitate the translation of preclinical discoveries into clinically applicable strategies.

Beyond their direct effects on core trophoblast cell behaviors, our study further identifies that miR-142-3p and RDH13 coordinately regulate the expression of key adhesion and migration-related molecules, namely CDH5 (VE-cadherin) (47), LFA-1 (integrin αLβ2) (48), and L-SELECTIN (CD62L) (49), at both transcriptional and translational levels. Consistent with their opposing roles in trophoblast function, the miR-142-3p inhibitor group exhibited significant upregulation of CDH5, LFA-1, and L-SELECTIN, while miR-142-3p mimics and si-RDH13 transfection led to marked downregulation of these molecules. This consistent expression pattern across mRNA and protein levels strongly supports that the miR-142-3p/RDH13 axis modulates trophoblast biology through the transcriptional regulation of these adhesion molecules, establishing a functional link between miRNA and downstream signaling cascades that govern cell behavior.

Notably, each of these molecules plays well-characterized roles in placental development and trophoblast function, which contextualizes their relevance to preeclampsia pathogenesis. CDH5, a key component of endothelial adherens junctions, is also expressed in trophoblast cells and contributes to cell–cell adhesion, migration, and the formation of the placental vascular network, which are processes critical for spiral artery remodeling (50, 51). LFA-1 and L-SELECTIN, classic leukocyte adhesion molecules, are increasingly recognized to mediate trophoblast cell interactions with the uterine decidua and vascular endothelium, thereby regulating trophoblast invasion and migration during placentation (52, 53). Dysregulated expression of these adhesion molecules has been previously implicated in preeclampsia: for example, reduced CDH5 expression in placental tissues correlates with impaired trophoblast invasion and increased vascular dysfunction in preeclamptic patients (54), while downregulated L-SELECTIN expression is associated with inadequate trophoblast-endometrial crosstalk (55). The alignment between our findings and these prior observations reinforces the biological significance of the miR-142-3p/RDH13-adhesion molecule regulatory axis. Specifically, we propose that miR-142-3p overexpression in preeclampsia suppresses RDH13, which in turn downregulates CDH5, LFA-1, and L-SELECTIN. This cascade ultimately impairs trophoblast cell adhesion, migration, and invasion, which were key processes disrupted in preeclampsia. Conversely, inhibiting miR-142-3p restores RDH13 expression and upregulates these adhesion molecules, rescuing trophoblast function. This mechanism is consistent with broader evidence that miRNAs regulate trophoblast behavior by targeting adhesion and cytoskeletal molecules (56), and extends this paradigm by identifying RDH13 as a critical mediator of these downstream effects. Fetal growth restriction is often comorbid with preeclampsia and shares core pathological features, such as impaired trophoblast invasion and placental insufficiency (57). The miR-142-3p/RDH13 axis regulates trophoblast proliferation, invasion, and vascular-related molecules (CDH5/LFA-1/L-SELECTIN), which are critical for fetal growth support. Collectively, these data suggest that the miR-142-3p/RDH13 axis modulates trophoblast function through coordinated regulation of CDH5, LFA-1, and L-SELECTIN, providing a mechanistic framework that connects miRNA dysregulation to impaired placentation in preeclampsia.

However, this study has several limitations. First, it relied primarily on *in vitro* experiments with HTR-8/Svneo cells, lacking *in vivo* validation using preeclampsia animal models to confirm the miR-142-3p/RDH13 axis’s pathophysiological relevance. Second, the direct binding between miR-142-3p and RDH13’s 3’UTR was not verified, and it also did not directly detect RDH13 mRNA and protein expression changes in miR-142-3p inhibitor-transfected cells. The functional dependency observed in rescue experiments supports a pathway-level interaction rather than a direct miRNA-target relationship, which should be addressed in future studies via dual-luciferase reporter assays or RNA immunoprecipitation (RIP) experiments. Third, clinical sample sizes were limited, and the diagnostic specificity of HEXB/RDH13 needs validation in larger, multi-center cohorts. Future research should use animal models to validate the axis’ in vivo function, confirm direct miRNA-target binding via dual-luciferase assays, verify the direct expression regulation of RDH13 by miR-142-3p through RT-qPCR and WB assays, expand clinical samples, and explore additional downstream molecules/pathways of the miR-142-3p/RDH13 axis to enrich preeclampsia’s mechanistic understanding and lay a foundation for the development of targeted therapies.

## 5 Conclusion

This study identifies HEXB and RDH13 as potential early biomarkers of preeclampsia diagnosis via bioinformatics and clinical validation. Functional and rescue assays confirm that miR-142-3p impairs trophoblast function in a partially RDH13-dependent manner, with RDH13 mediating the regulation of CDH5/LFA-1/L-SELECTIN axis. These findings identify a novel functional association between miR-142-3p and RDH13, providing potential diagnostic biomarkers and candidate targets for preeclampsia research.

## Data Availability

The datasets analyzed during the current study are available in the GEO repository: https://www.ncbi.nlm.nih.gov/geo/; accession numbers: GSE25906 and GSE10588.

## Footnotes

1
https://www.ncbi.nlm.nih.gov/gds

2
https://mirwalk.umm.uni-heidelberg.de/
