# Supplementary figures and images for: miR-142-3p regulates RDH13 to impair trophoblast function via regulating CDH5/LFA-1/L-SELECTIN axis: a novel mechanism and diagnostic/therapeutic for pre-eclampsia
Source: Front Med (Lausanne). 2026 Mar 25;13:1760916. doi: 10.3389/fmed.2026.1760916 (PMC13057265; doi:10.3389/fmed.2026.1760916)

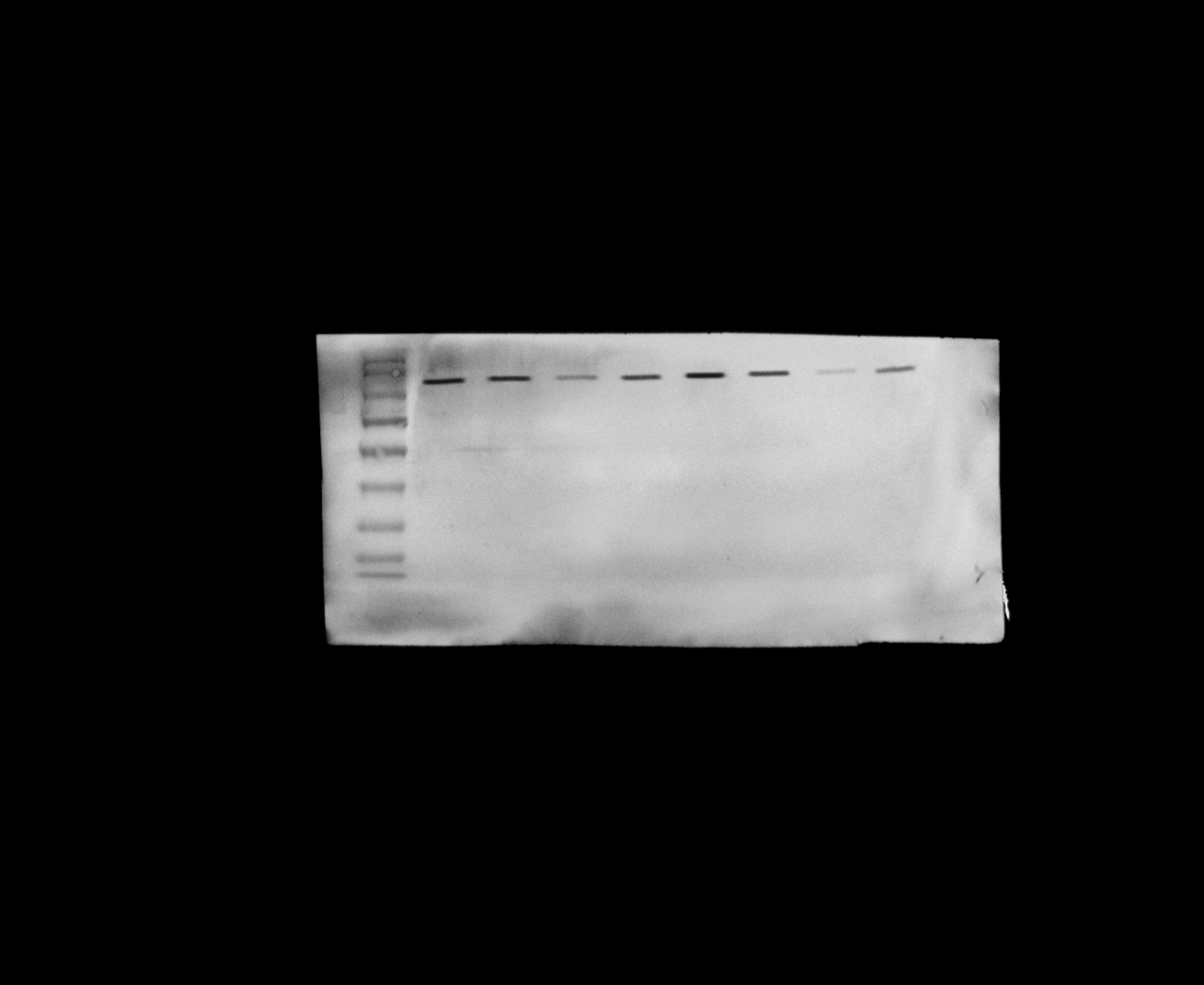

Supplement: Supplementary file 1 [file Image_1.TIF]

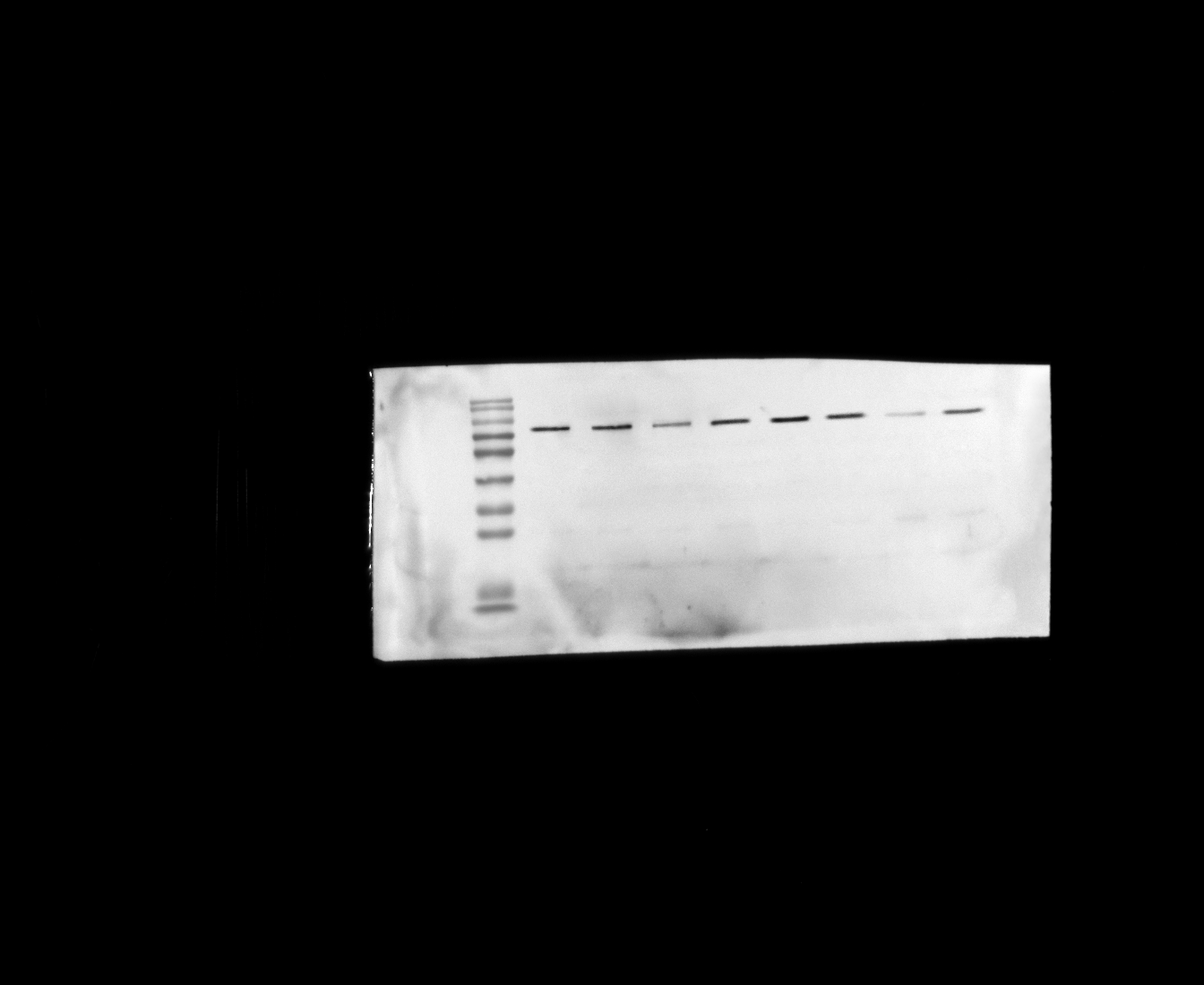

Supplement: Supplementary file 2 [file Image_2.TIF]

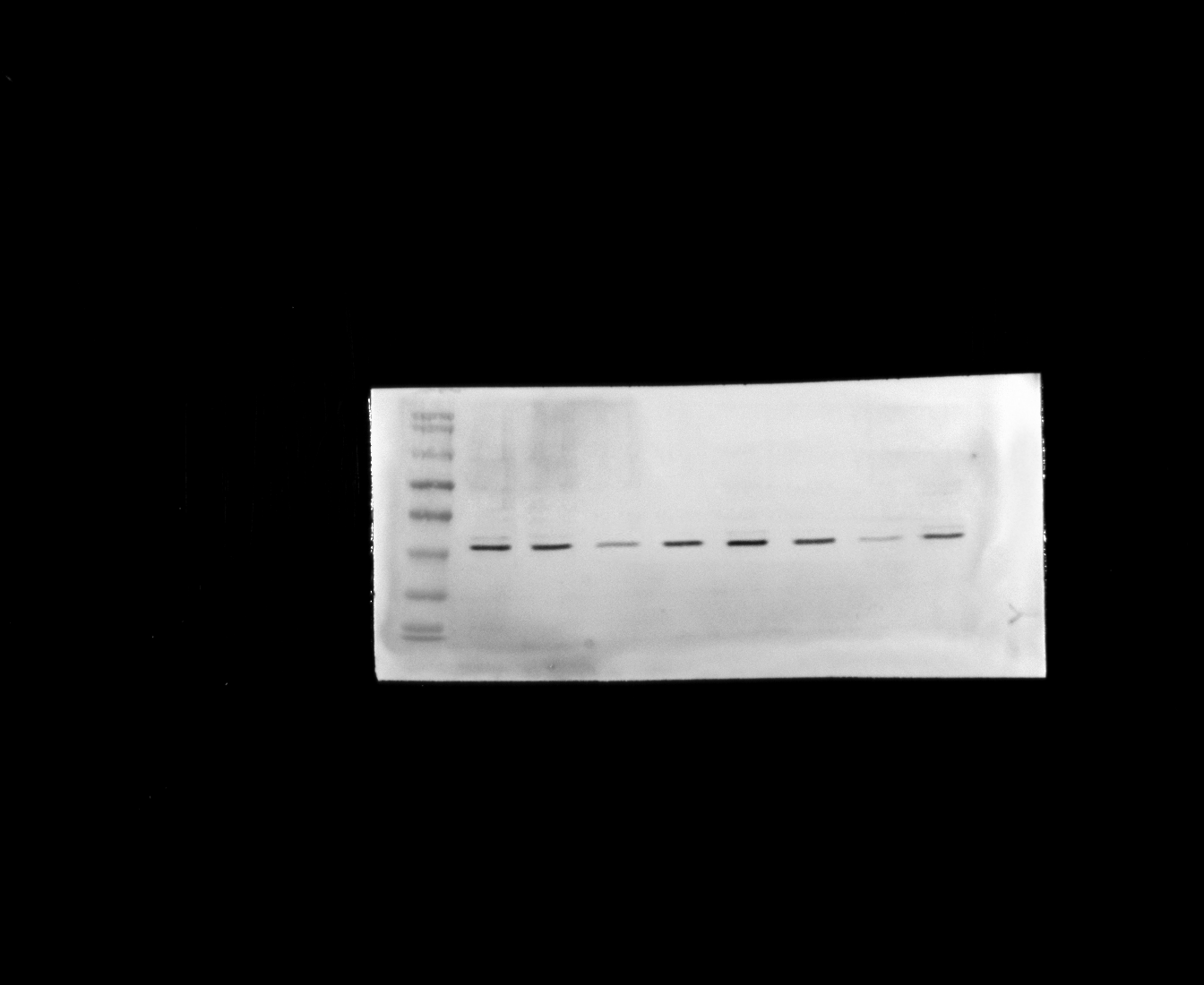

Supplement: Supplementary file 3 [file Image_3.TIF]

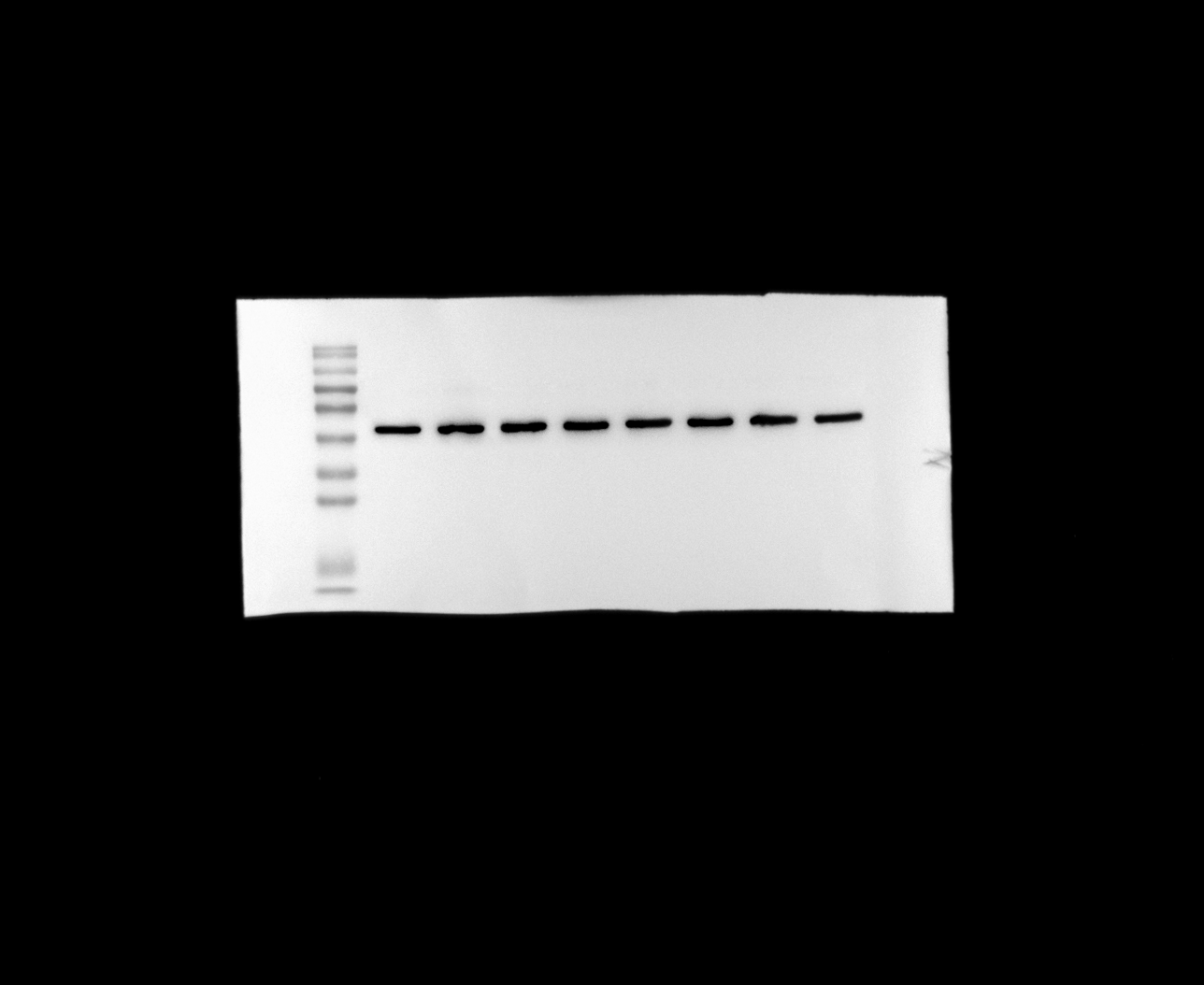

Supplement: Supplementary file 4 [file Image_4.TIF]
